# Replication concerns in sports and exercise science: a narrative review of selected methodological issues in the field

**DOI:** 10.1098/rsos.220946

**Published:** 2022-12-14

**Authors:** Cristian Mesquida, Jennifer Murphy, Daniël Lakens, Joe Warne

**Affiliations:** ^1^ Centre of Applied Science for Health, Technological University Dublin, Tallaght, Dublin, Ireland; ^2^ Human-Technology Interaction Group, Eindhoven University of Technology, Eindhoven, The Netherlands

**Keywords:** hypothesis testing, publication bias, statistical power, questionable research practices, Open Science practices, replicability

## Abstract

Known methodological issues such as publication bias, questionable research practices and studies with underpowered designs are known to decrease the replicability of study findings. The presence of such issues has been widely established across different research fields, especially in psychology. Their presence raised the first concerns that the replicability of study findings could be low and led researchers to conduct large replication projects. These replication projects revealed that a significant portion of original study findings could not be replicated, giving rise to the conceptualization of the replication crisis. Although previous research in the field of sports and exercise science has identified the first warning signs, such as an overwhelming proportion of significant findings, small sample sizes and lack of data availability, their possible consequences for the replicability of our field have been overlooked. We discuss the consequences of the above issues on the replicability of our field and offer potential solutions to improve replicability.

## 1. Introduction

Null hypothesis significance testing (NHST) is a method of statistical inference where the probability (*p*-value) of observed or more extreme data is compared against the hypothesis of null effect (i.e. null hypothesis). In the Neyman–Pearson approach to NHST, the observed *p*-value is compared with a pre-established alpha level rate (usually *α* = 0.05). If the observed *p-*value is smaller than the pre-established alpha level, the researcher can claim that statistical significance has been reached and act as if the null hypothesis were false^1^ with a maximum error rate of the alpha level. Statistical significance (i.e. *p* < 0.05) should not be confused with practical significance since it only means that the observed data are extreme enough such that an effect as extreme as, or more extreme than, has been observed would occur less than 5% of the time, if the null hypothesis was true [2]. One interesting observation is that over 90% of published studies using NHST in biomedicine and psychology reported significant findings (i.e. *p* < 0.05) [3–5]. Similarly, it has been observed that between 70% and 82% of published studies in sports science journals reported significant findings [6,7]. One conclusion that can be drawn based on these data is that researchers in these disciplines plan and design studies that usually reject the null hypothesis, because their studies examine predominantly true effects^2^ with high statistical power (henceforth, power).

However, it is unlikely that the high proportion of significant findings in these fields is solely due to high-quality research designs and testing true effects. One key fact that should render researchers skeptical about the replicability of prior findings is when a literature body produces more significant findings than expected, based on the power of the study designs [5,8] (see table 1 for the definition of replicability). For instance, while in psychology over 90% of published studies reported significant findings, the average power to detect a medium effect size has been estimated to barely reach 50% [16,17] or even lower [18,19]. An excess of significant findings is problematic and indicates that other factors play a role that bias the proportion of significant findings in the published literature. Three main factors identified in the literature are: publication bias, including reviewer bias and the file-drawer problem [8,11,12]; questionable research practices (QRPs), including HARKing and *p*-hacking [13–15,18] (see also [20,21] for researchers' degrees of freedom); and studies with underpowered designs [17,18,22,23], among others [24–26] (see table 1 for definitions). Together, these factors contribute to the probability that a published significant finding is actually a false positive and, consequently, the systematic presence of these issues in a literature body is likely to hinder its replicability.

**Table 1.** Definitions of key concepts.

*Excess of significance findings*
The phenomenon whereby a body of literature produces a higher percentage of significant findings than should be expected, given the average power of the design of these studies.
*Statistical power*
The probability of a statistical test rejecting the null hypothesis when it is false, i.e. the probability of obtaining a significant finding. It depends on the given effect size of interest, the chosen significance level and the number of participants tested [9].
*Replicability*
This refers to testing the reliability of a prior finding using the same methods and statistical analysis as in the original study but by collecting *new* data [10]. It differs from reproducibility in that the latter refers to testing the reliability of a prior finding using the same data and same statistical analysis.
*Publication bias*
This relates to publishing behaviours that give studies which find support for their tested hypotheses a higher chance of being published, as opposed to the publication of replication studies and non-significant findings. These behaviours include editors and reviewers selectively publishing studies with significant findings (i.e. review bias; [11]) and researchers deciding not to submit studies with non-significant findings (i.e. file-drawering) [12].
*Questionable research practices (QRPs)*
QRPs describe a set of research behaviours that can spuriously increase the probability of finding evidence in support of a hypothesis [13]. Some forms of QRPs are HARKing and *p*-hacking [13,14].
*HARKing*
A form of QRP that involves the *post hoc* formulation of the hypothesis after the results are known [15].
*p-hacking*
A form of QRP that exploits flexibility in data analysis to obtain significant findings [13]. Examples of *p*-hacking include optional stopping, the inclusion or exclusion of data on the basis of *post hoc* criteria, and multiple testing [13,14].

These aforementioned issues raise concerns about the credibility of scientific findings and sparked interest in replicability across scientific fields such as psychology and pre-clinical cancer biology [27–31]. One of the first attempts to systematically replicate study findings was the Open Science Collaboration Project [27], which set out to replicate 100 primary findings published in three high-impact psychology journals; strikingly, although 97% of the original studies reported significant findings, only 37% of the replication studies yielded a significant finding in the same direction as the original study. This project was followed by other replication attempts in psychology [32], social sciences [28] and economics [29], with replication rates of 54%, 62% and 61%, respectively. Despite these developments in other fields, replication studies are still very rare in sports science [33]. This might be in part not only due to the difficulties in conducting replication studies observed across disciplines [28,33,34], but also due to particular features of sports science research. Firstly, it is practically impossible to conduct replications of published studies that require long-term observations/interventions (e.g. multiple exposures to altitude training), expensive equipment and samples with unusual traits (e.g. elite athletes). Secondly, replication studies may require expertise that only a few researchers have, such as the study of motoneuron adaptations to resistance training by using high-density electromyography analysis [35]. Finally, limited availability of original raw data, inaccurate explanation of procedures or methods, and poor reporting practices in the original study hinder the assessment of replicability ([32,34]; see §2.3 for explanation). Before performing a large-scale replication project in sports science, it seems reasonable to first evaluate the extent to which methodological issues may influence the replicability of the published literature.

To date, few studies have investigated the presence of the aforementioned methodological issues in sports science [6,36–39]. Their findings have raised the first warning signs that our scientific field is likely to face a problem with replicability due to an overwhelming proportion of significant findings, small sample sizes and lack of research data availability [6,36–39]. However, the consequences of methodological issues such as publication bias, QRPs and studies with underpowered designs, which are known to increase the number of false positives in the published literature, have been overlooked. Therefore, the purpose of the current review is to discuss the potential consequences of these aforementioned methodological issues on the replicability of sports and exercise science findings, and offer potential solutions to combat this in the future. We hope that this review will encourage other researchers to examine the presence of these and other methodological issues in larger literature bodies, conduct replication studies where needed, and increase the adoption of Open Science practices, such as conducting *a priori* power calculations and making research data available to facilitate replicability.

## 2. Methodological issues

In line with previous findings in biomedicine and psychology [4,40], Büttner *et al*. [6] reported that out of 129 studies from sports and exercise medicine journals, 106 (82.2%) reported significant findings. For this percentage to be a true representation of the studies performed in the field, both the power and the proportion of true hypothesis tested must exceed 80% [8]. In other words, nearly all hypotheses that sports and exercise researchers test must examine a true effect, and either the effects investigated or the sample sizes used must be consistently large enough to achieve the desired power (i.e. greater than or equal to 80%). In the following sections, we discuss why 82% significant findings in the literature should be interpreted with caution.

### 2.1. Publication bias and questionable research practices

**Figure 1.**
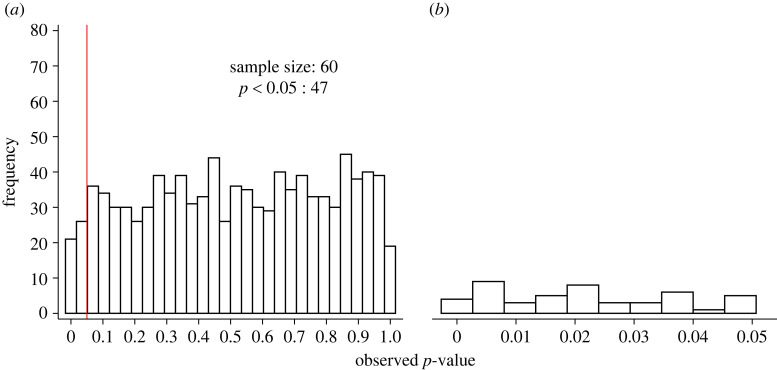
Distribution of *p*-values over: (*a*) [0–1] interval and (*b*) over [0–0.05] interval when the null hypothesis is true. One thousand *p*-values were generated for simulated comparisons with an unpaired *t*-test for statistical difference between two samples of 60 participants each. The red line denotes statistical significance at *p* < 0.05 and the number of significant *p*-values representing type I errors.

One way to objectively examine the reliability of a set of findings is to quantify the evidential value of a literature body [41]. Evidential value is determined by the number of studies examining true and false effects, the power of the studies that examine true effects, the frequency of type I error rates (and how they are inflated by *p*-hacking), and publication bias [42–44]. Fortunately, issues relating to the power of the studies, *p*-hacking and publication bias can be explored via the distribution of reported *p*-values [43,44]. For example, when the null hypothesis is true, *p*-values between a [0–1] interval should be equally likely in a two-sided hypothesis test regardless of the sample size, yielding a uniform distribution [42,44,45] (figure 1). In other words, when the null hypothesis is true, a *p*-value of 0.01 is just about as likely to be observed as a *p*-value of 0.9.

**Figure 2.**
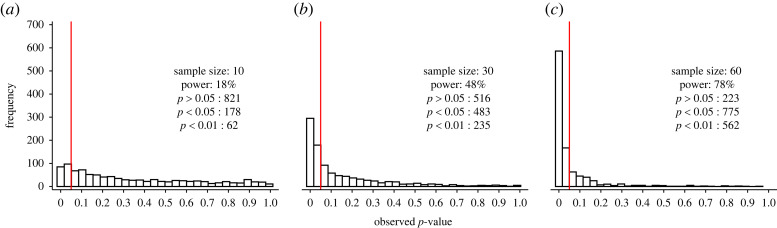
Power affects the distribution of *p*-values when the null hypothesis is false. One thousand *p*-values were generated for simulated comparisons with an unpaired *t*-test for each sample size. The number of *p*-values below 0.05 and 0.01, and above 0.05 are shown. The power is the percentage of simulations in which the *p*-value reaches significance (i.e. *p* < 0.05), given that the alternative hypothesis is true. The vertical red line denotes statistical significance at *p* < 0.05.

However, when the alternative hypothesis is true, the distribution of *p*-values becomes a function of power, and thus the study sample size and the true (but always unknown) effect size [45,46]. The sample size is therefore an important factor when evaluating the distribution of *p*-values in literature. Suppose there is a true effect between two populations with a Cohen's *d* effect size (effect size *d*) of 0.5 and we perform an unpaired *t*-test to test this difference in three different sample sizes (i.e. 10, 30 and 60 participants per group). As we can see in figure 2*a*, a sample size of 10 per group and a true effect size *d* of 0.5 yields a power of 18%, which means that out of 1000 replications, only 180 should be expected to reach statistical significance (in the long run), even though there is a true effect to be found. With a sample size of 60 participants per group, power is as high as 78%, meaning that 780 out of 1000 replications reach statistical significance in the long run (figure 2*c*). In studies with high power and where a true effect is examined, the likelihood of observing a small *p*-value (e.g. *p* = 0.01) is higher compared with a large *p*-value (e.g. *p* = 0.4) [45,46]. Moreover, as power increases even more, most of the *p*-values are below 0.01, and there are relatively fewer *p*-values between 0.01 and 0.05 (figure 2). For instance, while there are 235 *p*-values below 0.01 with a power of 48%, there are as many as 562 with a power of 78%. Consequently, the *p*-value distribution (in sufficiently powered study designs) follows a right-skewed distribution, where larger *p*-values become increasingly less frequent (i.e. it is a monotonically decreasing function) in unbiased literature—that is, in the absence of *p*-hacking and publication bias [47]. For this reason, the distribution of *p*-values can be used not only to determine whether a set of homogeneous studies investigates true or false effects, but it can also be used to estimate the average power of the set of studies. Altogether, it should be clear that the small sample sizes observed in sports and exercise science [36,39] may be a reason for concern, given the high proportion of significant findings that are observed [6,7].

**Figure 3.**
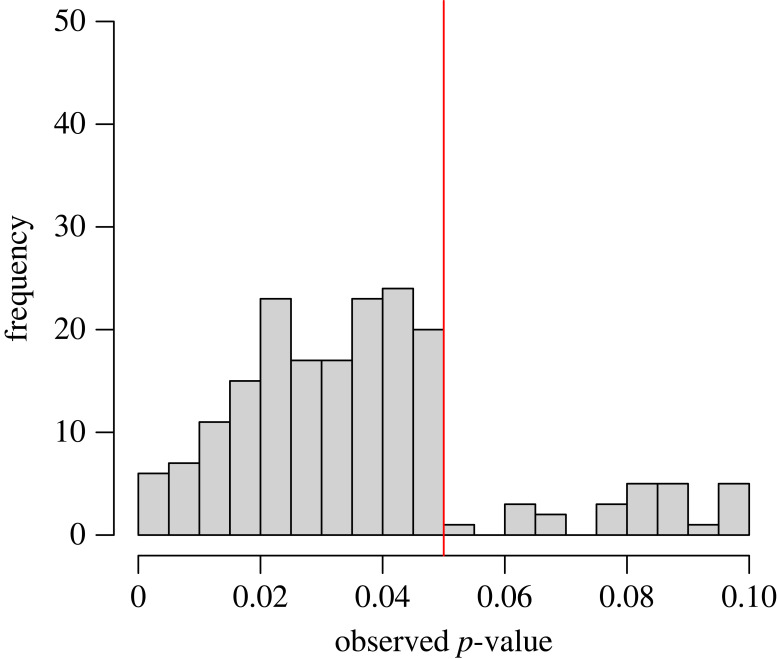
Distribution of *p*-values over [0–0.1] interval when the null hypothesis is true but in the presence of *p-*hacking. This would reflect the influence of collecting 10 participants and conducting an unpaired *t*-test after each addition until 100 participants are collected. The red line denotes statistical significance at *p* < 0.05.

While the above assumes an unbiased *p*-value distribution, one explanation for an excess of significant findings in a literature body that has been raised is publication bias and *p*-hacking [13,48,49].^3^ In the presence of publication bias (where non-significant findings are less likely to get published), researchers have incentives to explore *post hoc* analyses to find a significant *p*-value (i.e. *p*-hacking). If *p*-hacking occurs in literature, the distribution of reported significant *p*-values adopts different shapes [42]. For instance, when researchers resort to optional stopping (when the null hypothesis is true), the distribution of reported significant *p*-values is right-skewed (i.e. there will be a greater number of *p*-values between 0.04 and 0.05 than between 0.00 and 0.01; figure 3). The *p*-value distribution can also be used to examine a bias to publishing significant findings. The lack of a continuous distribution of *p*-values below the default alpha level of 0.05 and above this threshold indicates the presence of bias in favour of significant findings in the published literature (i.e. publication bias). Therefore, by examining the distribution of *p*-values, it can be determined whether published findings contain evidential value of a true effect, and the extent to which findings in the literature are affected by publication bias and/or *p*-hacking [43,44].

### 2.2. Power

**Figure 4.**
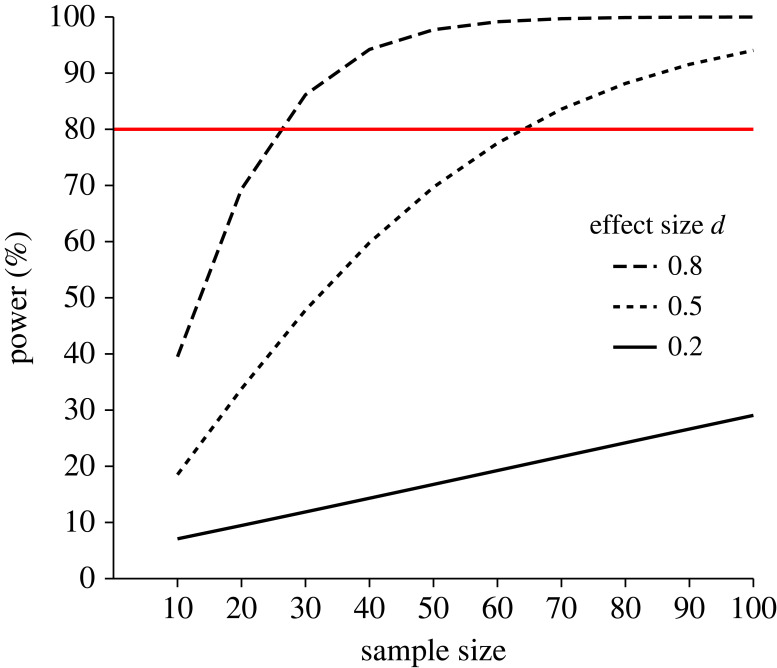
Power of an unpaired *t*-test, given a range of sample sizes and effect sizes. The red line denotes an adequate power of 80%.

In a Neyman–Pearson approach, researchers should use the NHST framework under the assumption of two conditions [50]. First, the null hypothesis should be plausible enough so that its rejection might be unexpected. Second, researchers should be willing to make a decision about a scientific claim for which the type I and type II error rates are adequately controlled. Researchers can limit the frequency of type I and type II errors by choosing the alpha level and conducting studies with high-power designs for effect sizes of interest, given that the type II error rate is defined as 1—power (the higher the power, the lower the type II error rate). To ensure that studies have well-powered designs, researchers should conduct pre-study power calculations for a given sample size and effect size of interest (figure 4). The value of this approach is discussed below.

#### 2.2.1. Estimating power in sports and exercise sciences

Power has direct implications on replicability because, from a frequentist standpoint, power is also described as the long-run probability of obtaining a significant effect when there is a true effect to be found [51]. To date, most researchers are familiar with Cohen's suggestion [52] that study designs should have at least 80% power. Hence, a study design is typically considered adequately powered if it finds a significant effect in 8 out of 10 replications when there is a true effect to be found (although one might argue that whenever feasible, a higher statistical power is desired). Moreover, according to Fisher [53], a good study should rarely produce a non-significant finding when the null hypothesis is false [53]. Therefore, if studies examining true effects are designed with high power, any researcher is more likely to find the same effect when replicating the same procedures with adequate power.

There is, however, concern that studies in sports and exercise science are not adequately powered for effects of interest. It is again worth highlighting the findings from two recent studies [6,36]; the high proportion (82.2%) of significant findings [6] and the small median sample sizes (*n* = 19) reported in the *Journal of Sports Sciences* [36] seem to indicate that, unless all examined effects are large, there might be relatively low power. As we will discuss in the following section, a median sample size of 19 is likely to yield underpowered designs, especially to detect small and medium effect sizes. The main implication of underpowered study designs is that the literature should be filled with a higher proportion of non-significant findings since the published studies would have a low probability of detecting the studied effect [54], but this is not the reality. To our best knowledge, only one study has assessed the power of a literature body in our field [55]. This study estimated the median observed power of 108 significance tests from 29 studies using fixed effect sizes based on Cohen's benchmarks [52]. The median observed power was 14%, 65% and 97% for small, medium and large effect sizes, respectively. Furthermore, moving beyond the median power and looking at individual studies, it was found that no studies had adequate power to detect small effect sizes, only 38% of studies had adequate power to detect a medium effect size and about 75% of studies had a power of at least 80% to detect large effect sizes. However, one limitation of this method was the use of fixed effect sizes based on Cohen's benchmarks, which are derived from effects observed in behavioural science [52]. It is uncertain whether Cohen's benchmarks accurately represent effect sizes observed in any given subfield of sports and exercise science [56–58]. For instance, Swinton *et al*. [58] conducted a Bayesian hierarchical meta-analysis to identify specific effect size benchmarks in strength and conditioning interventions, and reported that the benchmarks for small, medium and large effect sizes were 0.12, 0.43 and 0.78, respectively. Therefore, sports and exercise researchers should avoid the use of effect sizes based on Cohen's benchmarks for pre-study power calculations, and use specific effect sizes derived from meta-analysis [58] and, if possible, meta-analytical effect sizes adjusted for publication bias (and small-study effect) since they can also suffer from overestimation (see [59] for an example; see [60] for meta-analytic effect sizes compared with large-scale preregistered replications).

To further elaborate, we provide observed power estimates in our field using a typical effect size and sample size reported in previous research [36,61]. R code used for this power calculation is available at https://osf.io/y3482/. There is reason for caution because of the use of small sample sizes in our field [36,39]. Besides the small median sample size reported in the *Journal of Sports Sciences* (*n* = 19) [36], four biomechanics and sports science journals had a mean sample size (standard deviation, SD) of 21 (24), 15 (19), 32 (32) and 20 (22) (of 188 studies published in 2009 [39]). To see how sample size affects observed power, we will use an effect size *d* of 0.43, which has been reported to be the medium effect size benchmark for effects observed in 679 strength and conditioning intervention studies [58]. Suppose we conduct a study to find a true effect size *d* of 0.43 with a sample size of 20 for a paired *t*-test. This within-subject design would yield a power of 45%, implying that if 10 replications were to be conducted, only about five would find a significant effect. It is worth noting that for achieving 80% power, a sample size of 44 would be needed if the true effect size was *d* = 0.43. Small sample sizes might be appropriate if the true effect size being estimated is large enough to be reliably observed in such samples [22]; for instance, estimated effect sizes from strength and conditioning interventions might be much larger than those observed in sports performance research [56,57]. However, studies with small samples in combination with selective reporting of significant findings are susceptible to overestimate true effect sizes [62]. This means one should be cautious about the observed large effect sizes in the literature, if small studies are the sole source of these estimates [22]. Given the small samples reported in biomechanics and sports and exercise science journals [36,39], it might therefore be hypothesized that sports and exercise science faces a problem with underpowered designs, especially to detect small and medium effect sizes. However, it should be noted that within-subject designs have higher power than between-subject designs, given any effect size and sample size [63]. The extent to which within-subject designs can increase power compared with between-subject designs is given by the correlation between observations [63]. This is because correlation is typically positive and higher in within-subject designs compared with between-subject designs. Hence, the higher the correlation between observations, the higher the power achieved. Therefore, between-subject designs may potentially have even less power to detect the effect size of interest than the power estimated from a within-subject design. In the following section, we discuss the consequences of underpowered designs.

#### 2.2.2. Consequences of underpowered designs

**Figure 5.**
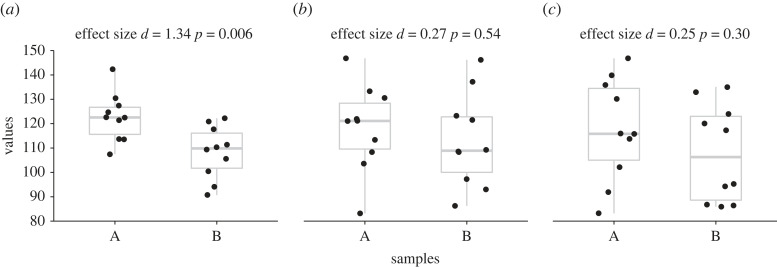
Small samples show substantial variation. To illustrate the variability of statistical outcomes derived from small samples, 6 samples of 10 values each were drawn at random from the same two populations, as in figure 2. The true effect size *d* between population A and B is 0.5. The estimated effect size *d* and *p*-value when sample pairs are compared are provided to demonstrate the variability of observed outcomes.

While low power in itself is caused by low sample size or small effect sizes*,* or both, the consequences of low power should be emphasized here. Firstly, underpowered designs are less likely to find a true effect even if the effect exists at the population level [17,64]. This is because small sample sizes contain a high sampling variance and therefore are less likely to not contain the true population parameters. This is demonstrated in figure 5, where even though there is a true difference between population A and B (i.e. effect size *d* of 0.5), two of three of the studies do not find a significant effect and thus commit a type II error.

Secondly, underpowered designs also increase the proportion of false positives in a literature body where there is publication bias [17,64], which is known as the *positive predictive value*. To see how this plays out, let us assume that 20 sports science studies within the same scope have an average power of 45%, as we have calculated previously assuming a total sample size of 20 and a medium effect size *d* of 0.43 for a paired *t*-test. In such a situation, approximately only 9 out of 20 studies (20 × 0.32) would find a significant effect even if all null hypotheses tested were false. The number of false positives with an alpha level of 0.05 would be 1 (20 × 0.05). Thus, the number of false positives relative to the total number of published significant findings is 10% (i.e. false positives/(false positives + correct hits) = 1/(1 + 9)). On the other hand, let us consider how things would play out if the average power in a set of 20 studies is 80% instead of 20%. In this case, the number of significant findings when there is a true effect to be found would be 16 (20 × 0.8). While the number of false positives would be the same (0.05 × 20 = 1), the proportion of false positives would be approximately 6% (1/(1 + 16)). Comparatively speaking, although an unbiased body of literature can only be achieved by publishing all study findings, irrespective of the *p*-value, the reliability of a literature body is higher when the power is 80% rather than 20%. In fact, a set of underpowered studies investigating the same effect and all reporting significant findings is so unlikely that the findings become literally improbable [17]. Suppose that a set of five studies with an average power of 45% has reported significant effects when the null hypothesis was false. The probability of all five studies finding a significant effect would be 1.85% (0.45^5^). Therefore, if the power observed in sports and exercise science studies is as low as hypothesized [36], we may expect an elevated number of false positives in sets of underpowered studies within the same scope. Given the observed high proportion of significant findings discussed [6], an elevated number of false positives seems a plausible explanation for a significant proportion of study findings published in this field.

**Figure 6.**
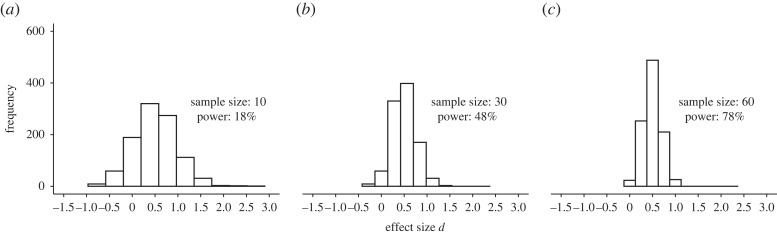
Sample size affects the estimation of the true effect size. Using the same data simulated as in figure 2, 1000 effect sizes were computed. The histograms show the distribution of effect sizes for three different sample sizes. As sample size increases, the estimated effect size becomes closer to the true effect size *d* of 0.5.

Thirdly, the effect size provided by a study with an underpowered design in the presence of publication bias is likely to be overestimated [22,27,28,65]. As observed in figure 5, when a significance test has low power due to a small sample size, a significant effect size will only be found when the effect size is relatively extreme [65,66]. However, when power is augmented by taking more observations, the estimated effect size becomes closer to the true effect size [65,66] (figure 6). For instance, both the Open Science Collaboration project [27] and the Social Science Replication Project [28] conducted replications with higher-power designs than the original studies; one of the main findings was that both replication projects observed that the mean effect size of the replicated studies was approximately 50% of that reported in the original studies [27,28]. Because of the observed small sample sizes reported in sports and exercise sciences [36,39], it is likely that the reported effect sizes are overestimated, further compounding the issue with low power. Another consequence is that if published effect sizes are overestimated and therefore do not reflect the true distribution of effect sizes, meta-analyses are compromised [60].

In addition, the overestimation of effect sizes is in itself a cause of concern when conducting pre-study power calculations [62,67]. The rationale for conducting a pre-study power calculation is to obtain an estimate of the sample size needed, given an effect size of interest and intended power. However, if the effect size used for the pre-study power calculation is overestimated, researchers may end up obtaining a smaller sample size and thus eventually achieving less power than intended [62]. This is especially problematic when studies use small sample sizes and in the presence of publication bias because only overestimated effect sizes will be published. For example, suppose a researcher wants to test the effect of a treatment on two independent samples and the true effect size *d*, which is unknown, is 0.5. The researcher wants to obtain the sample size required to achieve 80% power and uses an overestimated effect size *d* of 1.34 from a previous underpowered study (figure 5*a*). Thus, the researcher finds out that a sample size of 20 (i.e. 10 participants per group) is needed to achieve 80% power and detect an effect size *d* of 1.34 for an unpaired *t*-test. However, although the intended power was 80%, the overestimated effect size (i.e. effect size *d* = 1.34) yielded a true power of 19% (R code available at https://osf.io/y3482/). Thus, a researcher, who conducts a pre-study power calculation based on the likely overestimated effect size from an original small sample study, may end up designing a study which has less power than intended, and to compound the issue, the use of smaller sample sizes for a given power would ultimately yield overestimated effect sizes. This situation not only occurs when conducting pre-study power calculations based on effect sizes from previous studies with underpowered designs, but also when the effect size of interest is derived from a pilot study (i.e. follow-up bias; see [67]). Consequently, researchers should take care when choosing the effect size for a pre-study power calculation. As it is practically impossible to know the true effect size (and if it was known, there would be no need to collect additional data), researchers need to decide upon the expected effect size of interest; for example, based on the effect size estimated from a meta-analysis (and, if possible, adjusted for publication bias), or based on the effect size estimated from a previous study. However, in this case, researchers should use adjusting methods that account for the overestimation of the effect size due to small sample sizes and publication bias when conducting a pre-study power calculation [62,68]. A better approach is therefore to perform a power analysis based on the smallest effect size of interest [69].

**Figure 7.**
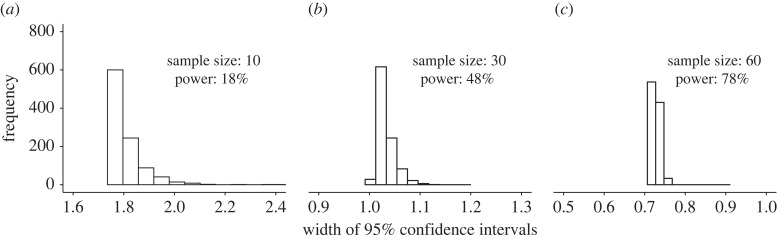
Sample size affects the estimation of the CIs. Using the same data simulated as in figure 2, 1000 95% CIs were computed. The histograms show the distribution of these 95% CI ranges for the same three different sample sizes. As sample size increases, both the range and the scatter of the CI decreases, reflecting increased power and greater precision from larger sample sizes.

Lastly, underpowered designs also decrease the precision of parameter estimates ([61,62]; figure 7). This is because the width of confidence intervals (CIs) around the parameter estimate depends on the SD and the number of observations. Thus, larger sample sizes produce smaller standard errors. The larger the CI around a parameter estimate, the less certain one can be that the estimate approximates the corresponding true population parameter [70]. As we can observe in figure 7, the width of a CI decreases as the sample size increases (which also increases the statistical power). Effect sizes and CIs obtained with larger samples are more precise than those obtained with smaller ones [70]. Similarly, it has been reported that out of a sample of 290 between-subject effect sizes *d* (Cohen's *d*) from five psychology journals, 83% of the effect sizes sampled had CI widths that were larger than the reported effect sizes and 26% were twice as large as the reported effect sizes [71]. As a consequence of the small sample sizes reported in sports and exercise science journals [36,39], it might be hypothesized that CI width might be larger than in other research areas with larger sample sizes, such as psychology, further compounding potential issues with the precision of our observations.

#### 2.2.3. Use of pre-study power calculations in sports and exercise science

**Figure 8.**
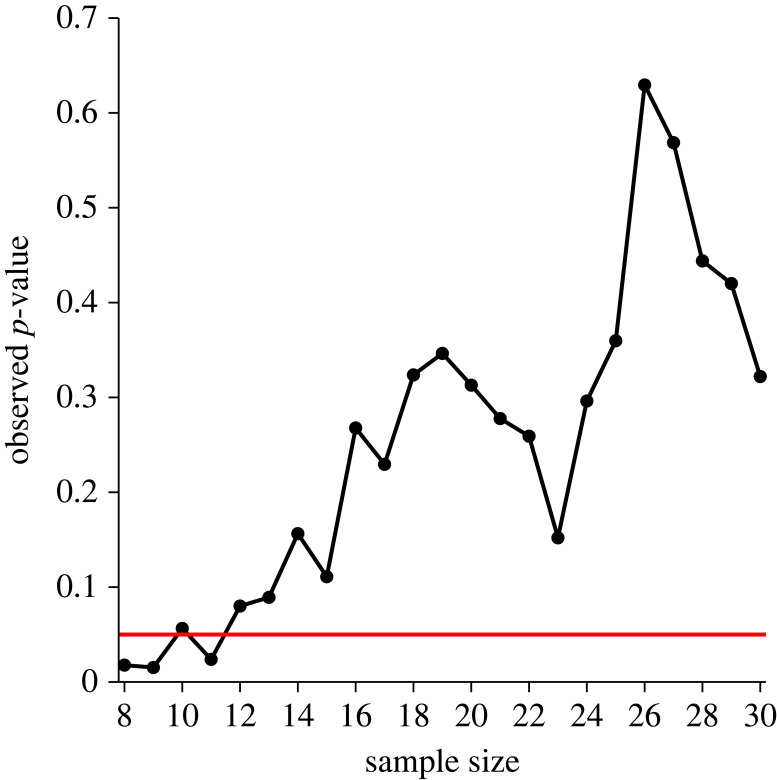
Illustrative simulation of *p*-values obtained by a researcher who continuously adds a participant to each of two sample groups and conducts an unpaired *t*-test after each addition. The horizontal red line denotes statistical significance at *p* < 0.05. Note that sample size refers to the number of participants per each of the two groups.

Despite the core importance of power in NHST, the use of pre-study power calculations is still scarce in sports and exercise science [36]. In 2000, it was reported that of 40 studies published in the *Journal of Science and Medicine in Sport*, no study included a pre-study power calculation [55]. More recently, Abt *et al*. [36] reported that only 10% of studies (12 out of 120) published in the *Journal of Sports Sciences* included such practice. Although this reflects an increased use of power analysis, it is clearly not a standard practice in our field. This is in marked contrast with the recent findings from Collins & Watt [72], who observed that 71% (152 out of 214) of psychologists self-reported to have used power analysis for sample size planning. There might be several reasons as to why pre-study power calculations are not standard practice in our field [70,72–74]. Firstly, researchers do not sufficiently understand this statistical concept and its importance in NHST [72]. This is reasonable to assume, as all studies (12 out of 12) from Abt *et al.* [36] that included pre-study power calculations failed to disclose full information on the statistical test to be conducted to detect the effect size of interest and four failed to include convincing rationale for why the given effect size was chosen. It has been argued that if researchers do not have sufficient understanding of power, they cannot be expected to successfully calculate and accurately report power analysis [72]. Secondly, researchers may rely on intuition, rules of thumb or prior practices, also known as heuristics, to determine study sample sizes [73,74]. For instance, of 187 psychology researchers, 45 (23%) mentioned some rule of thumb (e.g. 20 subjects per condition) and 41 (21%) based their sample sizes on the common practice in their field of research [73]. These practices might be a major concern especially in scientific disciplines using small sample sizes, and investigating small and medium effects sizes, because this combination would produce studies with underpowered designs, as previously discussed. Thirdly, a common practice among researchers to determine the number of participants is optional stopping [13,14]. This practice involves stopping collecting data earlier than planned because a significant effect was found (figure 8). This can occur in situations, for example, where a researcher who has already collected 30 observations per condition, and then tests for significance every 5 or 10 observations per condition [13]. However, such practice is considered a form of QRP because it leads to overestimated effect sizes and increased type I error rates [13]. Instead, sample size planning should be based on a goal of achieving adequate power or precise parameter estimates [64,70,74]. Therefore, given the scarce use of sample size planning based on power calculations and its lack of accurate reporting [36,72], it might be suggested that researchers in our field have a poor understanding of power and the consequences of low-power designs on type I error rate and effect sizes [22,23]. Furthermore, the scarcity of pre-study power calculations also suggests that sports and exercise researchers may rely on either heuristics or optional stopping for sample size planning. To improve, sports and exercise researchers might consider either consulting a statistician to help with the sample size justification for a new study, or educating themselves in best practices (for a review, see [72]).

### 2.3. Availability of research data

Availability of research data is a core scientific principle, not only because it contributes to cumulative science [75,76] and enables computational reproducibility ([73]; see [74] for a summary of studies on reproducibility of statistical results), but also because it enables researchers to design novel studies that help assess the replicability of published findings [10,34]. For instance, although the Reproducibility Project: Cancer Biology attempted to investigate the replicability of 193 experiments from 53 studies, only 50 experiments could be repeated [31,34]. Among others barriers identified to hinder replicability [34], only 4 out of 193 original studies reported key descriptive and statistical results needed to compute effect sizes, conduct pre-study power calculations and assess the success of a replication. Moreover, authors were unable to obtain these data for 68% of the experiments despite contacting the authors of the original studies. Data sharing therefore helps to design informative replication studies. Cumulatively, both poor reporting practices and lack of data sharing hinder the assessment of replicability.

#### 2.3.1. Data-sharing practices

Empirical data show that, in general, sports and exercise researchers are reluctant to engage in data sharing practices [37]. Indeed, Borg *et al*. [37] reported that only 13 of 299 studies published in 2019 in quartile-one sports science journals shared data. Yet, this is not surprising, given that only 5 of 286 studies stated that data was available upon request. The lack of data-sharing practices might be problematic for several reasons. Firstly, it has been reported that about 50% of published studies in psychology contain at least one inconsistent *p*-value and about 13% contain a grossly inconsistent *p*-value [77,78]. Secondly, the willingness to share research data has been related to the strength of the statistical significance and a higher prevalence of reporting statistical errors [79]. Interestingly, *p*-values in the interval between 0.03 and 0.05 (which are less likely to occur when there is a true effect to be found) were more common in papers that did not share data (16.7%) than in papers that did (9.1%). Thirdly, integrity surveys among researchers have revealed that the prevalence of QRPs was in the range of 33–51% [80,81]. More serious forms of misconduct, including fabrication and falsification of data or study findings, have been reported to range between approximately 2% and 4% [80,81]. In light of these findings, there is a clear need to adopt data-sharing practices that allow the research community to reproduce and replicate published study findings.

#### 2.3.2. Reporting practices

The *p*-value of a significance test is the main statistic used for deciding whether the null hypothesis can be rejected or not. However, researchers' poor understanding of the NHST often leads to the misconception that significance means a large effect, while no significance means a small effect or no effect [2,66,82]. In studies with underpowered designs, non-significant findings are hardly indicative of the absence of an effect, and with large sample sizes, effect sizes can be significant but practically irrelevant [69]. It has therefore been recommended to combine the *p*-value along with effect sizes and their CIs [66,83]. An effect size provides quantitative information about the magnitude of the relationship or effect studied, and its CI indicates the uncertainty of that measure by presenting the range within which the true effect size is likely to lie [65]. Furthermore, effect sizes and their CIs allow findings from several studies to be combined in the form of meta-analysis to obtain more precise effect sizes [65,76]. Despite this, the reporting of effect sizes and CI is usually omitted in sports and exercise science [38,55]. For instance, Speed & Andersen [55] reported that only 14% (4 out of 29) of studies published in the *Journal of Science and Medicine in Sport* reported effect sizes. Similarly, a more recent study observed that only 39% of studies published in the *Journal of Applied Biomechanics* in 2014 reported effect sizes [38]. These findings suggest an overreliance on *p*-values to interpret study findings despite the consequences of small sample sizes on the reliability of statistical results [22,23].

Besides the quantitative information, reporting effect sizes and their CI, or at least including sufficient information to calculate them, also contributes to improving the replicability of findings. For instance, researchers attempting to replicate an original study with a higher-power design will need the original effect size estimate to calculate the sample size of the replication study. Similarly, researchers might opt for a more conservative approach, which is to use the lower CI bound of the original effect size. Alternatively, researchers may use the precision-in-parameter-estimation method, which also requires CIs, to identify the minimum sample size that would ensure a precise estimate of the population parameter [64]. Therefore, the omission of reporting effect sizes and CI, along with failing to make raw data publicly available, may hinder any attempt at replication since other researchers might not be able to conduct a pre-study power calculation based on the original effect size or CI.

However, reporting only effect sizes and their CIs, and full information about the pre-study power calculations, might not be enough. With the aim of facilitating cumulative scientific knowledge through meta-analysis [75,76], and the use of other statistical methods such as *z*-curve/*p*-curve [44,84] or BUCSS to conduct power calculations adjusting for publication bias and uncertainty around parameter estimates [62], it has been suggested that besides sample size per condition, means, SDs and exact *p*-values, studies should also disclose *F*-ratio or *t*-statistics, the type of design, and the correlations between dependent observations for within-subjects designs [76], but it appears that this is rarely achieved. The compounding issues of poor reporting practices are easy to demonstrate with two examples; firstly, consider a within-subject design (i.e. pre versus post) in which a study reports means and SDs but not the within-subject effect size. Thus, researchers attempting to conduct a meta-analysis, and assuming the study meets the inclusion criteria, should use Hedges’ *g*_av_ effect size (effect size *g*_av_) from such a study [75]. However, these researchers may well not be able to calculate the effect size *g*_av_ (see supplementary file in [85]) because the correlation between observations is never reported. Alternatively, as long as means, SDs, number of observations, *t*-statistic and exact *p*-value are reported, researchers could use the user-friendly web application *within* [86] to estimate the correlation parameter and then calculate effect size *g*_av_. However, again *t*-statistics and exact *p*-values are often not reported. Finally, researchers may opt to ask the study authors for the correlation, the *t*-statistic or the raw data so that researchers can calculate it themselves. Yet, given the reluctance of sports and exercise science researchers for sharing data [37], one possible outcome is that researchers will not be able to get hold of this. Hence, researchers may have to discard the study due to poor reporting practices and lack of data sharing. Secondly, researchers attempting to conduct a pre-study power calculation using G*Power for a within-subject ANOVA will need the correlations between observations [87]. However, again this correlation is seldom reported. Taken together, these two hypothetical situations reflect some of the barriers that researchers have to overcome when attempting to conduct a meta-analysis or a pre-study power calculation.

Furthermore, the reporting of exact *p*-values and effect sizes not only informs about the statistical significance, direction and magnitude of an effect, but also can be used to answer meta-scientific questions (e.g. how replicable is a particular set of findings?) by performing a *z*-curve/*p*-curve analysis, a meta-analysis or a meta-meta-analysis. Addressing meta-scientific questions may require the analysis of large datasets (see [19,46,87–89] for examples). This can be facilitated by the use of software to scan, select and analyse large sets of published data, where statistical results should be machine readable. The ultimate goal is to enhance the ability of computers to automatically find and use the data, in addition to supporting its reuse by researchers (i.e. FAIR principles; see [90]). This can be facilitated by the adoption of common reporting practices, such as those recommended by the American Psychological Association (APA). Following APA standards, statistic test results should be reported in the following order: the *F*-ratio or *t*-statistic and degrees of freedom (in parentheses) followed by the *p*-value (e.g. *F*_1,35_ = 5.45, *p* = 0.001 or *t*_85_ = 2.86, *p* = 0.025). However, this is not a common standard reporting practice in sports and exercise science. Thus, adopting common reporting practices, such as APA's reporting recommendation, would facilitate machine readability and data usability, enabling the analysis of large sets of data containing *p*-values, effect sizes or CIs. The reporting of statistical results is key to replicating original studies, assessing the replication success and conducting additional statistical tests. However, the heterogeneity of our reporting practices in sports and exercise science makes a full evaluation of replicability in our field problematic, to say the least.

### 2.4. Future recommendations for sports and exercise science: adoption of Open Science practices

As a consequence of the above practices [17,22,23,36,70] and their effect on replicability rates reported by replication projects [27,28,30,31], Open Science practices are slowly being adopted within the research ecosystem. Open Science practices refer to a set of behaviours that enable research to be reproduced and replicated, with the aim of improving the reliability of study findings [70,91]. These practices may be especially important in research fields that reward publication of significant findings from studies with low-power designs and exploiting, either intentionally or not, researchers' degrees of freedom [13,21,92]. We herein suggest a series of Open Science practices that could be adopted by researchers and journals to improve the replicability in our field [70,93,94].

One practice is preregistration, which was conceived to mitigate QRPs by preventing HARKing and by reducing the risk of *p*-hacking via restricted flexibility in study design and data analysis [91,94]. In preregistered studies, authors register the protocol of their hypothesis, methods and analysis plan before data collection. Consequently, preregistered studies have been observed to produce smaller effect sizes than non-preregistered studies due to the likely absence of publication bias and QRPs [95]. However, preregistration alone may still not be enough to prevent publication bias [96,97]. Alternatively, Registered Reports are considered a more effective format against publication bias [8,91,98,99]. For instance, Scheel *et al*. [8] found that 96% of non-registered studies reported significant findings compared with 44% of Registered Reports. In a Registered Report, one submits a detailed plan of the research questions, hypotheses, methodology and analysis to a scientific journal for review prior to collecting data. Once a Registered Report is accepted, the journal agrees to publish the study if the quality-control criteria are met, regardless of the study finding. However, to date, only five sports and exercise science journals offer the Registered Report format, namely, *Journal of Experimental Physiology, Human Movement Science*, *Science and Medicine in Football* [100]*, Psychology of Sport and Exercise, *and* Reports in Sport and Exercise and Journal of Sports Sciences* [101]. Another practice that should be increasingly adopted is the use and reporting of pre-study power calculations for sample size planning to assure that studies are conducted with adequate power, given the effect size of interest [70,74]. In addition, low availability of research data reinforces the importance of sharing data including raw data, materials and code in public data repositories (e.g. Open Science Framework, Dryad Digital Repository and Zenodo), and improving the transparency and quality of reporting practices [70,91]. Sharing research data alongside a manuscript increases the transparency of the research process because it allows both reviewers and readers to verify the statistical results and therefore increase the reliability of the presented findings. Finally, sports and exercise researchers should conduct replications where needed and feasible [27–29,31,102–104]. Replication provides diagnostic evidence about a finding and allows for exploring the boundaries of studied effects, and ultimately, the progression of science by confronting the existing understanding with new evidence [10,32,54,105]. Despite the core importance of replicability, very few replication studies have been attempted in sports and exercise science [33]. In this regard, it is worth mentioning a current collaborative replication project in the field attempting to conduct close replications of original study findings [106].

## 3. Conclusion

Based on previous findings in other research areas [17,23,27,28,40] and similarities to our own discipline [6,33,36,37], several methodological issues, such as a high proportion of significant findings, studies with underpowered designs and inaccurate reporting practices, cast serious doubts about the replicability of sports and exercise science findings [6,33,36,37]. Firstly, there might be an excess of significant findings, given the high percentage of significant findings reported [6] and the observed power estimates we have provided. This excess may indicate the presence of other factors such as publication bias, QRPs and studies with underpowered designs that can increase the number of false positives and should be specifically investigated in future studies. Secondly, the small sample sizes reported in several biomechanics and sports and exercise science journals may also be a cause of concern, especially in studies using between-subject designs, for several reasons [17,22]. Small samples are likely to yield underpowered designs, which are known to increase the proportion of false positives and false negatives, produce overestimated effect sizes, and decrease the precision of parameter estimates (i.e. wide CIs). Thirdly, there is clear evidence that most studies do not report enough statistical results, such as effect sizes, CI, *F*-ratios, *t*-statistics and degrees of freedom, which directly impact the ability to evaluate methodological quality effectively. Altogether, although there is evidence indicating that our field is likely to face a problem with replicability, we acknowledge that the power estimates provided herein (based on a sample size of *n* = 19 [36] and an effect size *d* of 0.43 [58]) might not be representative of the field and should be interpreted with caution. Furthermore, sports and exercise science literature on this topic is very scarce and future studies should therefore systematically examine the presence of the aforementioned methodological issues. Yet, the evidence presented herein indicates that there is clear room for improving our research standards and highlights the importance of increasingly adopting Open Science practices in sports and exercise science research.

## Data Availability

The R code used for simulations, figures and power calculations is available on the Open Science Framework repository: https://osf.io/y3482/ [107].

## Footnotes

1 In the Neyman–Pearson approach to NHST, data are used to make decisions about how to act [1]. Researchers who rely on this approach should be interested in deciding to at least tentatively act as if one of the possible hypotheses is true. Thus, when researchers either ‘accept’ or ‘reject’ a hypothesis, they do not aim to communicate any belief or conclusion about the tested hypothesis but rather a basic statement that the observed data corroborates the tested prediction, or not.

2 Authors refer to true effects when there is an effect at the population level, which would be known if researchers could collect data from the entire population of interest.

3 Studies with unpowered designs and inflated type 1 error rates in a body of literature where researchers selectively publish significant findings increases the positive predictive value (the probability that a significant result in the literature is a false positive), which contributes to an excess of significant findings in the scientific literature.
